# Exploring the G-Quadruplex Formation of AS1411 Derivatives

**DOI:** 10.3390/molecules30081673

**Published:** 2025-04-08

**Authors:** Pedro Lourenço, David Moreira, André Miranda, Jéssica Lopes-Nunes, Izamara Maocha, Tiago Santos, Pedro L. Ferreira, Fani Sousa, Artur Paiva, Carla Cruz

**Affiliations:** 1 RISE-Health, Department of Medical Sciences, Faculty of Health Sciences, University of Beira Interior, Av. Infante D. Henrique, 6200-506 Covilhã, Portugal; pedro.afonso.amaro.lourenco@ubi.pt (P.L.); jessicalonu@hotmail.com (J.L.-N.); izamaocha@gmail.com (I.M.); fani.sousa@fcsaude.ubi.pt (F.S.); 2 RISE-Health, Department of Chemistry, Faculty of Sciences, University of Beira Interior, Rua Marquês d’Ávila e Bolama, 6201-001 Covilhã, Portugal; david.moreira@ubi.pt (D.M.); andre.miranda@ubi.pt (A.M.); pedroflf@live.com.pt (P.L.F.); 3 R&Di Division, Instituto de Soldadura e Qualidade, Av. Prof. Dr. Cavaco Silva, 33, Taguspark, 2740-120 Oeiras, Portugal; tiagoaasantos@hotmail.com; 4 Coimbra Institute for Clinical and Biomedical Research (iCBR), Faculty of Medicine, University of Coimbra, 3000-548 Coimbra, Portugal; artur.paiva@chuc.min-saude.pt; 5 Ciências Biomédicas Laboratoriais, Instituto Politécnico de Coimbra, ESTESC-Coimbra Health School, 3046-854 Coimbra, Portugal; 6 Unidade Funcional de Citometria de Fluxo, Centro Hospitalar e Universitário de Coimbra, Praceta Mota Pinto, 3000-075 Coimbra, Portugal; 7 Department of Chemistry, University of Beira Interior, Rua Marquês d’Ávila e Bolama, 6201-001 Covilhã, Portugal

**Keywords:** aptamers, AS1411 derivatives, G-quadruplex, DNA, nucleolin

## Abstract

AS1411 is a G-quadruplex (G4) aptamer that binds tightly to nucleolin (NCL) on the cell surface and has shown strong anticancer effects. However, this aptamer is highly polymorphic, presenting different types of G4s, which may hinder its preclinical application. Several modifications have been made to decrease the polymorphism of this aptamer. In this work, we designed six AS1411 derivatives by substituting guanine with thymine in the central linker and modifying the number of thymines either in the linker itself and/or at both ends of the sequence. The G4 formation, stability, and NCL binding were evaluated by several biophysical techniques and computational and cell studies. Overall, a decrease in polymorphism of G4-forming sequences compared to AS1411 is observed by size exclusion chromatography (SEC) and circular dichroism (CD) spectroscopy in the presence of potassium salt. The melting experiments reveal a higher ability of the derivatives without thymine at both sequence ends to form a G4, consistent with the G4H score predictions. Additionally, it is possible to conclude that deletions of T in the central core increase the ability to form G4. Moreover, the AS1411 derivatives bind NCL with high affinity (*K*_D_ values in the 10^−9^ M range), particularly the sequences with only thymine modifications in the central linker. In silico studies reveal structural insights and demonstrate that AS1411 derivatives interact with NCL, establishing multiple interactions with the different domains, thereby further supporting the experimental findings. By using a lung cancer cell line with high cell surface NCL expression, we evaluate the internalization and uptake of AS1411 derivatives, identifying the derivative-lacking thymines in the central core as the ones with the highest internalization and cellular uptake.

## 1. Introduction

Aptamers are short nucleic acid sequences that can adopt specific secondary and tertiary structures, enabling them to selectively and effectively bind to a wide range of targets with high affinity and specificity [1,2]. Their unique binding properties have made aptamers strong candidates for the development of innovative biomedical applications, including therapeutic agents, diagnostic tools, and targeted drug delivery systems [3,4,5]. The versatility of aptamers stems from their ability to be engineered and recognize various molecular targets, such as proteins, small molecules, and even entire cells, making them valuable assets in precision medicine [6,7,8].

AS1411 is a 26-mer guanine-rich DNA aptamer that has gained considerable attention in cancer research due to its specificity and therapeutic potential [9,10]. It functions as an anti-NCL agent, targeting NCL, a multifunctional protein that is overexpressed on the surface of cancer cells and plays a pivotal role in key cellular processes, including ribosomal RNA maturation, ribosomal DNA transcription, ribosome assembly, and RNA transport, and it also regulates the cell cycle, proliferation, and apoptosis [11,12,13]. Its aberrant expression in tumorigenic tissues makes it a compelling target for anticancer strategies, with AS1411 being one of the most extensively studied NCL-targeting aptamers [14,15].

The specificity and efficacy of AS1411 can be attributed to its ability to form a G4 structure, a non-canonical nucleic acid structure formed by sequences rich in guanines [16,17]. It arises when four guanine bases interact, forming a square planar structure known as a G-quartet, where each guanine within the quartet participates in hydrogen bonding, acting as a donor at its Watson–Crick edge and an acceptor at its Hoogsteen edge [18,19], forming a stable three-dimensional structure with monovalent cations like potassium (K⁺), and assuming different topologies (parallel, antiparallel, or hybrid) depending on the sequence and environmental conditions [20,21].

Given the promising potential of AS1411 and its inherent structural versatility, numerous modifications have been explored to enhance its performance [9]. These modifications may involve alterations to its nucleotide sequence, incorporation of non-natural bases, or conjugation with other moieties to improve stability and bioavailability [22,23,24,25,26,27,28]. Such advancements aim to optimize the therapeutic efficacy of AS1411, broaden its applications in cancer treatment, and pave the way for the development of aptamer-based therapeutics [29,30,31]. However, one study has been carried out to get detailed structural information on this aptamer, proving to be highly polymorphic, folded into multiple, essentially mono- and bimolecular G4 structures [32]. To reduce the conformational polymorphism of AS1411, and to exploit AS1411 as an active targeting agent for multifunctional nanosystems in anticancer strategies, we have designed a set of AS1411 derivatives with the insertion of thymine at each end of the sequence and modifications on the T central core, also a conjugate with a suitable reporter group providing special physicochemical features. The conformational behavior of AS1411 derivatives has been investigated using CD and UV spectroscopy, FRET-melting, PAGE, and SEC-HPLC to analyze their structure formation and stability. Additionally, docking and molecular dynamics simulations, fluorescence titrations, confocal microscopy, and flow cytometry have been employed to assess their interactions with NCL in silico and in vitro.

## 2. Material and Methods

### 2.1. Oligonucleotides and Reagents

The oligonucleotides used in this study are listed in Table S1 (Supplementary Materials) and were purchased from Eurogentec (Seraing, Belgium) and Eurofins (Luxembourg City, Luxembourg). Stock solutions were prepared with Milli-Q water and stored at −20 °C. When needed, oligonucleotide annealing was performed by heating the samples to 95 °C for 5 min and then quickly cooling them on ice.

The buffers used were K10 (10 mM KCl, 90 mM LiCl, 10 mM lithium cacodylate (LiCaCo) at pH 7.2); K20 (20 mM KCl, 80 mM LiCl, 10 mM lithium cacodylate at pH 7.2); K100 (100 mM KCl, 10 mM LiCaCo at pH 7.2). The solutions used in this work were prepared using ultrapure-grade water obtained from a Milli-Q system from Millipore (Burlington, MA, USA),

The designed sequences were analyzed using the G4Hunter v.2.0 web tool (https://bioinformatics.cruk.cam.ac.uk/G4Hunter/ (accessed on 1 October 2024)) [33].

PhenDC3, Thioflavin T (ThT), and *N*-methyl mesoporphyrin IX (NMM) were purchased from Sigma Aldrich (St. Louis, MO, USA) and Santa Cruz Biotechnology (Dallas, TX, USA) (Table S2), and the stocks were prepared in DMSO.

### 2.2. Förster Resonance Energy Transfer (FRET) Assays

#### 2.2.1. FRET-Melting Competition (FRET-MC)

The FRET-MC assay was conducted as previously described [34] using a CFX Connect™ Real-Time PCR instrument (Bio-Rad, Hercules, CA, USA). Experiments were performed in 96-well plates with a working volume of 25 µL per well (each containing 0.2 µM of the F21T fluorescent oligonucleotide, either with or without 0.4 µM of the G4 ligand (PhenDC3) and 3 µM competitors. All solutions were prepared in K10 buffer, and each condition was tested across three plates.

The instrument was programmed to record FAM emission while increasing the temperature stepwise by 0.5 °C per min from 25 °C to 95 °C. The resulting curves were normalized and fitted using a Boltzmann model in OriginPro2021 software Version 9.8 (OriginLab, Northampton, MA, USA) to determine the melting temperature (*T*_m_). The Δ*T*_m_ was calculated as the difference in *T*_m_ of F21T in the presence of a competitor compared to its absence with PhenDC3.

To classify and quantify the competitive effects of the tested oligonucleotides, the S Factor was calculated using Equation (1).

(1) $$S\;factor=\frac{\Delta Tm\;of\;F21T\;with\;competitors}{\Delta Tm\;of\;F21T\;alone}$$

Strong competitors have an S factor value near 0, while ineffective competitors maintain an S factor close to 1.

#### 2.2.2. Isothermal-FRET (iso-FRET) Assay

The isothermal competition experiment was conducted in 96-well plates (ref.650901; Greiner Bio-One GmbH; Kremsmünster, Austria) as previously described [35]. The samples were pre-denatured and were prepared in K20 buffer. Briefly, 5 μL of competitor (2.5 μM) was mixed with 5 μL of 37Q (1 μM) and 10 μL of PhenDC3 (2.5 μM). The mixture was incubated for 5 min before adding 5 μL of F22 (100 nM).

Before the fluorescent measurement, the plates were incubated at 37 °C for 24 h. The fluorescence signal was then recorded using a GloMax® Explorer Multimode Microplate Reader (Promega, Madison, WI, USA). The F factor was calculated using OriginPro2021 software (OriginLab, Northampton, MA, USA) with Equation (2), and fluorescence intensities were plotted. This F value parameter, derived from fluorescence intensities (*FI*), indicates the impact of a competitor on F22-37Q hybridization.

(2) $$F\;value=\frac{{FI}_{Competitors}-{FI}_{F22+37Q\;duplex}}{{FI}_{F22}-{FI}_{F22+37Q\;duplex}}$$

The global alignment analysis function of the NCBI database (https://blast.ncbi.nlm.nih.gov/Blast.cgi (accessed on 5 December 2024); National Center for Biotechnology Information, Maryland, USA), based on the Needleman–Wunsch algorithm [36], was used to calculate the CF factor (Equation (3)) and assess potential complementarity between competitors and F22 (Table S1). The analysis was conducted using the NUC4.4 scoring matrix (https://ftp.ncbi.nlm.nih.gov/blast/matrices/NUC.4.4 (accessed on 5 December 2024)). A gap penalty of 10.0 was chosen to minimize excessive fragmentation by effectively preventing large insertions and deletions, which are rare in conserved genomic regions. The extension penalty of 0.5 was chosen to allow for moderate gap elongation, ensuring that biologically relevant insertions and deletions are not excessively penalized.

(3) $$CF\;factor=\frac{Numbers\;of\;base\;pairs\;expected\;in\;(F22+Competitor\;duplex)}{Length\;F22\;}$$

### 2.3. Circular Dichroism (CD) Spectroscopy

CD spectra were obtained in a Jasco J-815 spectropolarimeter (Jasco, Tokyo, Japan) with a Peltier-type temperature controller (model CDF-426S/15) at 20 °C, over a range of 200–320 nm in a 10 mm quartz cell (Ref. 115B-10-40, Hellma Analytics, Müllheim im Markgräflerland, Germany), with a scan speed of 100 nm/min, 1 nm bandwidth, and 1 s integration time over 4 accumulations. Oligonucleotide sequences were prepared at a concentration equivalent to 0.8 absorbance and annealed in LiCaCo. Titrations were performed by adding increasing amounts of KCl directly in the cuvette, with an incubation time of 5 min.

CD-melting was also performed at the final titration point (100 mM KCl) by monitoring the denaturation process at 260 nm. The data were recorded over a range of 20–95 °C, with a heating rate of 2 °C/min, and spectra were read every 5 s of equilibration time. Data were converted into folded fraction (*θ*) plots according to Equation (4).

(4) $$\theta =\frac{CD-{CD}_{\lambda }^{min}\;}{{CD}^{max}-{CD}_{\lambda }^{min}\;}$$

where *CD* is the ellipticity of the monitored wavelength at each temperature, and *CD^min^* and *CD^max^* are the lowest and highest ellipticities, respectively. Data were fitted to a Boltzmann distribution using OriginPro2021 (OriginLab Corporation, Northampton, MA, USA), and the melting temperature was determined.

### 2.4. UV Spectroscopy

All UV–Vis experiments were conducted using a P9 UV/Visible double-beam spectrophotometer (VWR, Radnor, PA, USA). Absorbance spectra were recorded in a 10 mm quartz cuvette (Ref. 115B-10-40, Hellma Analytics, Müllheim im Markgräflerland, Germany) over a 200–350 nm range, with a scan rate of 300 nm/min, data intervals of 0.5 nm, and an integration time of 0.05 s. Automatic baseline correction was applied throughout. All oligonucleotides were kept at a concentration corresponding to 0.8 abs. Data were analyzed using OriginPro2021 (OriginLab Corporation, Northampton, MA, USA).

#### 2.4.1. Thermal Difference Spectra (TDS)

Oligonucleotide solutions were annealed in 10 mM Lithium Cacodylate (LiCaCo) supplemented with 100 mM of KCl (K100 buffer), and spectra were recorded at 20 °C to represent the folded state. The temperature was then increased to 95 °C, and after 15 min of equilibration, a second spectrum was acquired for the unfolded state. TDS were obtained by subtracting the unfolded spectrum from the folded one.

#### 2.4.2. Isothermal Difference Spectra (IDS)

Oligonucleotide solutions were annealed in 10 mM LiCaCo buffer (pH 7.2) following established protocols. The first spectrum was recorded at 25 °C in the absence of potassium, corresponding to the unfolded state. KCl was then added directly into the cuvette to reach a final concentration of 100 mM. After 30 min of equilibration, a second spectrum was recorded to capture the folded state. Spectra were corrected for dilution, and IDS were determined by subtracting the unfolded spectrum from the folded one.

### 2.5. Non-Denaturing Polyacrylamide Gel Electrophoresis (PAGE) Analysis

PAGE was used to assess G4 formation and molecularity in the sequences. Oligonucleotides were prepared at 1 μM in Milli-Q water and annealed as described in K100 buffer. After annealing, 10 μL of 50% sucrose was added to each sample, and 20 μL of the oligonucleotide solution was loaded into the gel (20% polyacrylamide gels supplemented with 100 mM KCl). Oligonucleotide markers (containing sequences of 9, 15, 21, 30, 60, and 90 nucleotides) were run in parallel for reference. Electrophoresis was carried out in a Mini-Protean II vertical electrophoretic cell (Bio-Rad, Hercules, CA, USA) powered by a PowerPac™ system (Bio-Rad, Hercules, CA, USA) at 110 V for 150 min at 4 °C. Before sample loading, the gels were pre-run at 100 V for 30 min at 4 °C.

Following the run, the gel was incubated for 30 min under gentle agitation with N-methyl mesoporphyrin IX (NMM) and Thioflavin T (ThT), both G4-specific ligands that fluoresce upon binding. The gel was then washed and stained with 1× SYBR Gold for 15 min under continuous agitation, and visualization was performed using a ChemiDoc™ XRS imaging system (Bio-Rad, Hercules, CA, USA).

### 2.6. Size Exclusion Chromatography (SEC)

SEC experiments were performed with an ÄKTA Pure 25 system (Cytiva, Uppsala, Sweden) equipped with a YMC-Pack Diol-200 column (4.6 × 300 mm; 3 μm Dihydroxypropyl-modified silica particles, 200 Å pore size; ref. DL20S03-3046WT; YMC, Kyoto, Japan) at 20 °C, following previously reported protocols [37]. Before the first injection, the column was equilibrated with at least three column volumes (15 mL) of K100 buffer.

Oligonucleotides were annealed in elution buffer (K100) and eluted at a flow rate of 0.150 mL/min. Eluted species were detected by absorbance at 260 nm, and the resulting chromatograms were normalized (scaled 0–1) and plotted using OriginPro2021 (OriginLab, Northampton, MA, USA).

### 2.7. Fluorescence Spectroscopy

Fluorescence titration experiments were performed using high-precision quartz Suprasil cuvettes (3 × 3 mm light path, Ref. 105–251–15-40, Hellma Analytics, Müllheim im Markgräflerland, Germany) and a FluoroMax-4 spectrofluorometer (Horiba, Kyoto, Japan).

Oligonucleotides were prepared at 100 nM and annealed in K100 buffer, as previously described. Titrations were carried out by stepwise addition of molar equivalents of NCL protein (domains 1,2 and 2,3), allowing 5 min of equilibration at each step before measurement.

Fluorescence spectra were recorded between 655 and 800 nm, with excitation at 647 nm. Each spectrum was acquired with an emission slit of 5 nm and an excitation slit of 5 nm, and when fluorescence signals were too high, the excitation slit was set to either 2 nm or 3 nm. To enhance signal reliability, three consecutive scans were averaged.

Fluorescence data were processed to generate fraction-bound DNA (α) plots using Equation (5)

(5) $$\alpha =\frac{I-I_{\lambda }^{free}\;}{I_{\lambda }^{bound}-I_{\lambda }^{free}\;}$$

where *I* is the fluorescence intensity at 664 nm for each DNA/Protein ratio and *I*^free^ and *I*^bound^ are the fluorescence intensities of the free and bound DNA, respectively. Additionally, a Hill saturation binding function was applied to fit data points, using OriginPro2021 (OriginLab Corporation, Northampton, MA, USA), according to the following Equation (6):

(6) $$\alpha =\frac{{\left[DNA\right]}^{n}\;}{\;K_{D}+{\left[DNA\right]}^{n}}$$

where *n* is the Hill constant, which describes the cooperativity of ligand and probe binding, *K*_D_ is the apparent equilibrium constant, and [*DNA*] is the concentration of the oligonucleotide sequence.

### 2.8. Molecular Docking and Molecular Dynamics (MD) Simulations

The three-dimensional structure of AT14, AT14T, and NCL RBD23 was predicted using the AlphaFold 3 server (https://alphafold.ebi.ac.uk (accessed on 26 November 2024)) [38] due to the absence of an experimentally determined structure. The most representative models of the structures were selected based on AlphaFold 3 confidence metrics, including predicted local distance difference test (pLDDT) scores and predicted aligned error (PAE) values, as well as structural consistency [38]. The 3D structure of NCL RBD12 (PDB: 2KRR) [39] was obtained from Protein Data Bank (PDB) [40]. All 3D structures were optimized using the dock prep tool from ChimeraX 1.9. The predicted models of AT14 and AT14T were subsequently refined using an AMBER14SB_OL15 force-field through fully solvated 500 ns molecular dynamics simulations, employing GROMACS 2022.3.cuda12, with the appropriate parameters [41]. The final equilibrated structure obtained from the simulation was used as a structural scaffold for further molecular docking and MD studies.

Then, molecular docking experiments were performed to predict the binding site of AT14 and AT14T in NCL models RBD12 and RBD23. The experiments were conducted by using AutoDockTools 4.2 (ADT4.2), with appropriate parameters [41], and the resulting molecular docking poses were ranked based on the predicted binding free energy. The most relevant conformers were selected for further analysis by MD simulations.

MD simulations were performed to evaluate the stability and dynamic behavior of the complexes. Simulations were conducted using GROMACS 2022.3.cuda12 with the AMBER14SB_OL15 force-field. Each system was solvated in an octahedral box containing K^+^ ions and TIP3P water molecules. Energy minimization was carried out using the steepest descent algorithm, followed by equilibration under NVT and NPT ensembles for 100 ps each. The production phase was run for 200 ns with a 2 fs time step. Temperature and pressure were maintained at 300 K and 1 bar using the modified Berendsen thermostat and Parrinello–Rahman barostat, respectively. Long-range electrostatic interactions were handled using the Particle Mesh Ewald (PME) method, and constraints on hydrogen bonds were applied via the LINCS algorithm. The trajectories were analyzed to assess structural stability, binding interactions, and conformational changes over time. All molecular figures and hydrogen bond analysis were performed using UCSF ChimeraX 1.9.

### 2.9. Confocal Fluorescence Microscopy

The human non-small cell lung cancer cell line (H1299) was obtained from the American Type Culture Collection (ATCC) and cultured in 75 cm^2^ *T*-flasks at 37 °C in a humidified atmosphere containing 5 % CO_2_. The cells were cultured in RPMI medium (Sigma-Aldrich, St. Louis, MO, USA) supplemented with 10 % FBS, 1 % streptomycin/penicillin antibiotic, 2 mM L-glutamine, 10 mM HEPES, and 1 mM sodium pyruvate.

For fluorescent microscopy, H1299 cells were seeded at 10 × 10^4^ cells/mL in treated μ-slide eight-wells (IBIDI, Gräfelfing, Germany), with 200 μL of medium per well, and incubated overnight to allow cell adhesion.

To assess NCL-mediated internalization, cells were pre-treated with an anti-NCL antibody (PA3–16875, Invitrogen, Waltham, MA, USA) to saturate the cell’s surface protein [42]. For that, cells were incubated with the primary antibody (1:100 dilution) for 2 h at room temperature, followed by a 24 h incubation with 1 μM Cy5-labeled sequences (annealed with KCl). Wells without antibody treatment were included to observe normal sequences’ internalization. After incubation, cells were stained with a Hoechst 33342 nuclear probe (2 μM final concentration from a 20 mM stock) for 15 min, washed with PBS, and imaged using a Zeiss AxioObserver LSM 710 microscope (Oberkochen, Germany), with 405 nm and 633 nm laser excitation for Hoechst 33342 and Cy5, respectively. Similar experiments were performed with sequences annealed in the absence of KCl to determine the influence of salt in the sequences’ internalization by the cancer cells.

### 2.10. Flow Cytometry

H1299 cells were seeded in 12-well plates at a density of 50 × 10^4^ cells/well and incubated overnight to allow cell adhesion. The cells were stimulated for 24 h with the sequences AT14, AT14T, and AT14-T2, at a final concentration of 1 µM, both in the presence and absence of 100 mM KCl. Following the incubation period, the wells were washed with PBS, and the cells were trypsinized and resuspended in PBS. Then, the cells were analyzed using a BD FACS Canto™ II flow cytometry system (BD Life Sciences, Franklin Lakes, NJ, USA) to evaluate the uptake of the aptamers. The cellular uptake of the sequences was monitored through Cy5 fluorescence, while non-specific staining, doublets, and debris were excluded from the analysis.

### 2.11. Statistical Analysis

Statistical analyses were performed using GraphPad Prism 9.0.0 (Boston, MA, USA), using analysis of variance (ANOVA) followed by Tukey’s Multiple Comparison Test. Data were considered statistically significant when *p* < 0.05.

## 3. Results and Discussion

Due to its great potential in therapy and diagnostics, AS1411 is a strong candidate for further research. However, its polymorphic structure complicates the understanding of its mechanism of action [32]. To overcome this, several studies have attempted to stabilize the aptamer by modifying its sequence or attaching nanomaterials, which can enhance its structural rigidity, improve target binding, or increase its resistance to enzymatic degradation [16,43,44]. In this study, we focus on altering specific bases within AS1411, specifically the central thymine linker, and/or by adding thymines at both 5′- and 3′-ends, and evaluating various modified candidates.

### 3.1. Design and G4 Formation Assessment by the Aptamer Derivatives

#### 3.1.1. FRET-MC and iso-FRET

**Figure 1:**
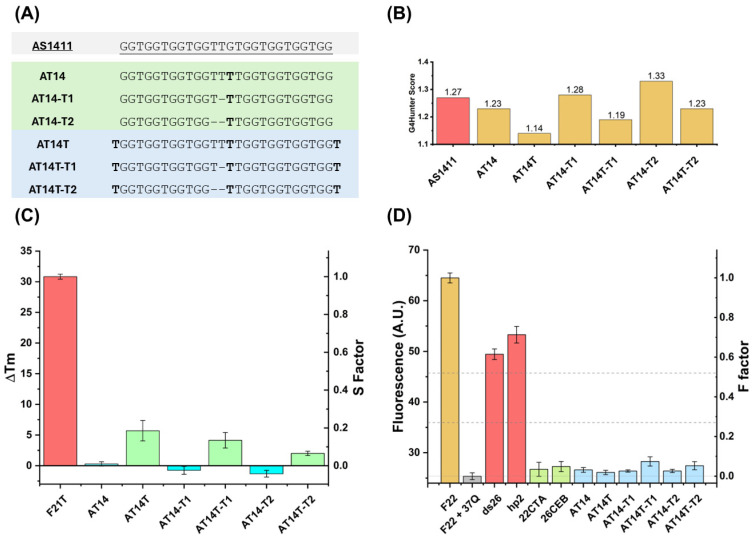
Design, algorithm evaluation, and high-throughput validation of AS1411 DNA aptamer derivatives as G4 forming sequences. (**A**) Design of aptamer derivatives by substitution of G nucleotide for T nucleotide into position 14, deletion of T from the central core (marked in green), and addition of T in 5′- and 3′-ends of the DNA sequences (marked in blue). (**B**) Prediction of G4 forming ability using the G4Hunter score value. Initial validation of appetence for designed aptamer derivatives assembles into a G4 structure using high-throughput methods like (**C**) FRET-MC and (**D**) iso-FRET (negative and positive controls labelled in red and green, respectively, and Oligos in study marked in blue).

Aiming to enhance its properties and reduce the polymorphism presented by the parental AS1411 DNA aptamer, and inspired by the modifications made by Phan’s group [27], new derivatives were designed. Considering the primordial sequence, it is possible to observe a quasi-palindromic image on the primary sequence (GGT)_4_ repeats, separated by TG nucleotides; considering the 5′ to 3′ and 3′ to 5′ senses). Thus, to build a perfect palindrome, and to favor a single G4 fold, we performed a substitution of G14 of AS1411 by a T in the same position (denominated by AT14; Figure 1A), similar to the strategy employed by Phan’s group [27]. Then, knowing that a direct relationship between thermal stability and a bulge/linker size exists, modifications were performed by varying the thymine linker size (deleting 1 and 2T; AT14-T1 and AT14-T2, respectively; Figure 1A). After designing the last three aptamer derivatives, thymine nucleotides were added to both 5′- and 3′-ends of the DNA sequences (Figure 1A), to prevent the stacking of G4s at the 5′ and 3′ ends, which could result in aggregation [45].

Subsequently, the G4Hunter score was accessed (Figure 1B) and revealed that the G–T substitution leads to a decrease in the score, and as expected, the deletion of 2 thymines in the central thymine core induces an increase in scores. In the same way, the addition of thymines at both ends of constructs results in a G4H score decrease.

After this evaluation, the first step was to determine whether the modifications made to the sequences affected G4 formation and, if so, how. To address this, several biophysical assays were employed to decipher the mechanistic/structural behaviors of modifications. We started with high-throughput methods, namely FRET-MC and iso-FRET, which are based on the PhenDC3 ligand competition among an oligonucleotide reported and the competitor (oligonucleotide in study). The results, presented in Figure 1C,D, respectively, show that all the aptamer derivatives can form G4 in vitro. Interestingly, FRET-MC techniques reveal a higher ability from the constructs without thymines in the ends (low Δ*T*_m_ and S-Factor) to form a G4, consistent with the G4H score predictions. Additionally, it is possible to conclude that deletions of T in the central core increase the ability of G4 formation. In the case of iso-FRET, no measurable differences were observed amongst oligonucleotide derivatives, as evidenced by the F-Factor.

#### 3.1.2. CD Studies

**Figure 2:**
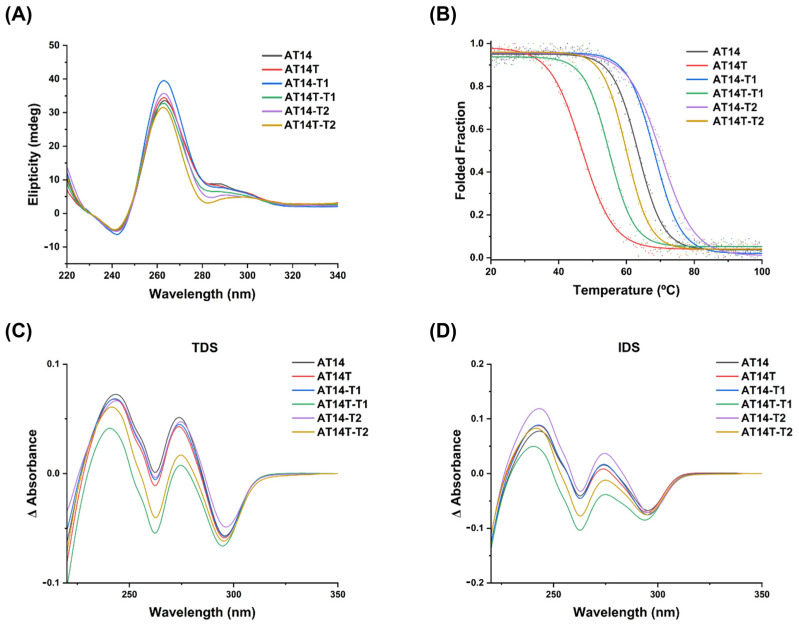
Spectroscopic analysis of AS1411 derivatives in K100 buffer. (**A**) CD spectra of the derivatives, showing their structural features. (**B**) CD-melting curves and *T*_m_ values, assessing their thermal stability. (**C**) TDS analysis, obtained by subtracting the spectra of the heat-denatured sequences from those under optimal conditions. (**D**) IDS analysis, comparing spectra in the presence and absence of KCl to evaluate potassium-induced structural changes. The concentrations of the sequences were adjusted to maintain an absorbance of 0.8.

To continue validating the G4 formation and structure topology (parallel, anti-parallel, or hybrid), CD studies were carried out. Firstly, a KCl dependence experiment was performed to understand how it affects the G4 formation and stability. The results evidenced that all the derivatives form G4 with a parallel topology (glaring by the typical positive bands at 260 nm and a negative one at 240 nm) [46], and the KCl stabilizes and induces a G4, explained by the ellipticity increase on spectra (Figure 2A).

By comparing the spectra at lower concentrations, we can conclude that AT14-T1 and AT14-T2 are the ones that form the G4 structure more easily, since the typical bands appear well defined with just 1 mM KCl. All the other sequence spectra only display the well-defined typical bands at 10 mM KCl (Figure S1).

Moreover, CD-melting was used to determine the thermal stability of the sequences (Figure 2B). From the obtained melting temperatures (*T*_m_; Table S3), we can conclude that the insertion of a thymine at each end of the sequences highly decreases their thermal stability (Δ*T*_m_ = −16.6 °C in AT14T; Δ*T*_m_ = −13.4 °C in AT14T-T1 and Δ*T*_m_ = −10.2 °C in AT14T-T2). It is described that the addition of a thymine nucleotide to the 5΄- and 3΄-ends of the sequence prevents the stacking of G4, resulting in higher-order structures that are usually highly stable [45,47]. Regarding the modifications on the thymine central core, the remotion of nucleotides leads to more stable sequences (Δ*T*_m_ = +4.9 °C and Δ*T*_m_ = +6.8 °C when removing two or one thymines from the central core, respectively). This behavior was already described for other AS1411 derivatives [27].

#### 3.1.3. UV Spectroscopy Analysis

To further corroborate the results obtained from CD experiments, a UV spectroscopy analysis was performed, specifically, using thermal difference spectra (TDS) and isothermal difference spectra (IDS).

Both methods rely on comparing the absorbance spectra of the unfolded and folded states, with the key distinction being the mode of denaturation, where TDS use thermal denaturation, while IDS assess structural changes in the absence of KCl cations, imperative for G4 formation. A characteristic TDS spectrum for a G4 structure typically displays bands around 245 nm, 273 nm, and 295 nm [48]. As shown by the TDS results (Figure 2C), these bands were present in all sequences, indicating that G4 structures were formed. Similarly, IDS produced a spectra difference with the same profile across all sequences (Figure 2D), showing peaks at the same wavelengths as TDS. This suggests that G4 formation occurs upon the addition of KCl, reinforcing the structural stability of these sequences under near-physiological conditions.

#### 3.1.4. PAGE and SEC Analysis

**Figure 3:**
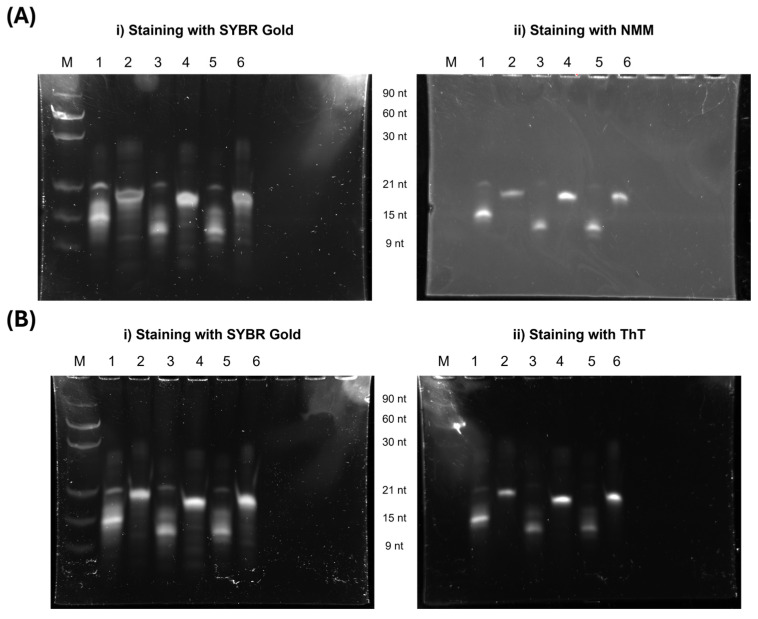
PAGE analysis of AS1411 derivatives in K100 buffer. The gel was supplemented with 100 mM KCl and run at 120 V for 150 min to assess the structural behavior of the derivatives. Lane assignments: M—oligonucleotide ladder; 1—AT14; 2—AT14T; 3—AT14-T2; 4—AT14T-T2; 5—AT14-T1; 6—AT14T-T1. (**A**) Gel stained with i) SYBR Gold and ii) NMM and (**B**) stained with i) SYBR Gold and ii) ThT, both to assess G4 formation by the derivatives. All sequences were kept at 1 µM.

G4 studies were further investigated using PAGE analysis, where gels were stained with G4-specific ligands to assess G4 formation and examine migration differences influenced by the presence of T residues. As shown in Figure 3, all lanes, except for the ladder, display strong fluorescence upon staining with NMM and ThT, indicating that both ligands bind to the G4 structures formed by the sequences.

Interestingly, sequences without thymines at the 5′ and 3′ ends (Lanes 1, 3, and 5) exhibit two fluorescence bands, a strong band around 15 nt and a fainter one around 21 nt. This suggests the presence of multiple G4 conformations or potential higher-order structures.

In contrast, sequences with thymine bases at both the 5′ and 3′ ends (Lanes 2, 4, and 6) migrate more slowly than those without these bases. This result suggests that the absence of terminal thymines favors the formation of a more compact G4 structure, which migrates faster in the gel. The presence of thymine at both ends may influence folding dynamics, potentially stabilizing a different conformation or inducing structural rearrangements that affect electrophoretic mobility.

Additionally, to confirm the previous data, SEC-HPLC experiments were conducted. Contrary to PAGE experiments, SEC results did not evidence major differences among aptamers, except for AT14T-T1, which revealed a later elution peak (Figure S2). Also, peak quantification showed that the deletion of thymine increases the bimolecular portion, in the case of the AT14 aptamer derivatives set, and the opposite effect was observed in the AT14T data set (Figure S3).

### 3.2. Interaction Studies Between Aptamer Derivatives and NCL

NCL is a multifunctional protein involved in various cellular processes and its overexpression in many cancer cells makes it an attractive therapeutic target. The AS1411 aptamer is known for its high affinity for NCL and has shown promise in therapeutic applications due to its ability to selectively bind this protein. This next section focuses on examining the AS1411 derivatives to explore their interactions with NCL.

#### 3.2.1. Fluorescence Titrations

**Figure 4:**
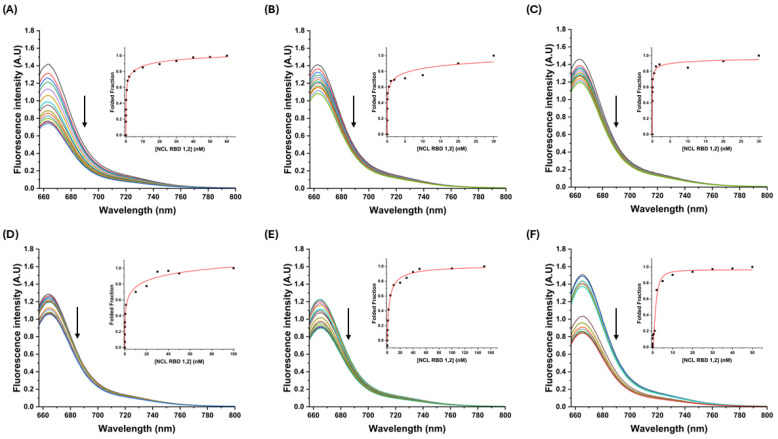
Fluorescence titration of pre-folded (**A**) Cy5-AT14, (**B**) Cy5-AT14-T1, (**C**) Cy5-AT14-T2, (**D**) Cy5-AT14T, (**E**) Cy5-AT14T-T1, and (**F**) Cy5-AT14T-T2 at 100 nM, with increasing concentrations of NCL RBD1,2 ranging from 0 to 150 nM.

Regarding the fact that AS1411 is a well-known aptamer towards NCL, the affinity of the derivatives used in this work toward NCL was checked through fluorescence titrations using Cy5-modified sequences (Figure 4 and Figure S4). The titrations of the pre-folded G4 sequences with NCL RBD1,2 and NCL RBD2,3 were fitted using the Hill model when possible.

The apparent equilibrium constants (*K*_D_) are presented in Table S4. Based on the results, the modifications can lead to very significant variations in the affinity that sequences have for NCL. Starting on NCL RBD1,2 which are the most studied domains in terms of interactions with AS1411 aptamers [49,50], it is possible to observe that the sequences that have modifications only on the thymine linker present the highest affinity, namely AT14-T2 and AT14, with *K*_D_ values of 0.01 × 10^−9^ M and 4.6 × 10^−9^ M, respectively.

AT14T, which has thymine on each side, presents the lowest affinity (*K*_D_ = 8.34 × 10^−9^ M), followed by AT14T-T1 and AT14T-T2. In both cases, the affinity increases when the thymine on the linker decreases. In the case of NCL RBD2,3, the behavior is slightly different. The removal of nucleotides on the linker did not affect the affinities toward NCL RBD2,3 in such a considerable way since AT14 has a *K*_D_ of 0.08 × 10^−9^ M, while AT14-T1 and AT14-T2 have a *K*_D_ of 2.16 × 10^−9^ M and 5.37 × 10^−9^ M, respectively. However, the insertion of the thymine on each side completely changed the interaction of the sequences with NCL RBD2,3. AT14T and AT14T-T2 seem to not have a measurable interaction with NCL RBD2,3, since the variations of the obtained fluorescence are slightly erratic, and it was not possible to fit the obtained data into the Hill model. On the other hand, AT14T-T1 maintains a similar affinity of the sequences without the thymine insertions (*K*_D_ = 0.03 × 10^−9^ M).

Then, these modified sequences also showed that it is hard to predict how a modification on an aptamer can influence the affinity to its target, since virtually every single nucleotide on an aptamer can be part of an important interaction between the aptamer and its target. Even the nucleotides on the loops or bulges of the G4 structures can be important to these interactions, as shown by MD simulations on similar sequences [26]. It is also not easy to conclude whether favoring a single G4 conformation can improve or not improve the affinity towards the target, as previous interaction studies of AS1411 with NCL have revealed *K*_D_ values in the pM range [51], and among our derivatives, only AT14-T2 shows a *K*_D_ value close to the reported range.

#### 3.2.2. Computational Studies

To predict the molecular interactions and binding sites of the AT14 and AT14T structures with NCL RBD12 and RBD23, molecular docking experiments and molecular dynamics (MD) simulations were performed. Initially, the 3D structures of AT14 and AT14T were constructed, followed by a fully solvated 500 ns MD simulation. Final snapshots were extracted from the MD runs and analyzed.

**Figure 5:**
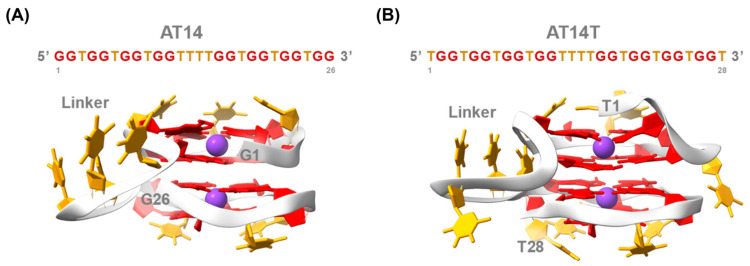
G4 structures of (**A**) AT14 and (**B**) AT14T. Guanine residues are shown in red, thymine residues in orange, the DNA backbone in light grey, and K^+^ in purple. Additionally, the terminal bases are explicitly labeled.

The results are consistent with previous studies on similar sequences, such as AT11 and its derivatives, confirming a four-layer G4 structure [26,27,28,51]. Both AT14 and AT14T adopt a topology comprising two parallel-stranded propeller-type subunits connected by a central linker comprising four thymine residues (Figure 5, Figures S5A and S6A). RMSD plots from MD simulations indicate that both structures exhibit high stability (Figures S5C and S6C). Furthermore, MD simulations revealed that in AT14, the most flexible nucleotides were those within the linker region (T12, T13, T14, and T15) (Figure S5B,D). It is worth noting that, in the AT14T G4, in addition to the linker nucleotides, T1 and T4 displayed significant mobility, contributing to the formation of a thymine cap that stabilizes the top two G-tetrads (Figure S6B,D). However, a potential drawback of this structural arrangement may be its reduced flexibility, as demonstrated by PAGE experiments, which could hinder its ability to adapt and interact efficiently with NCL. Another hypothesis, supported by CD-melting results, suggests that thymine residues contribute to structural destabilization, possibly leading to the formation of a distinct G4 with lower thermal stability. Overall, these results were anticipated and are strongly aligned with recent data, further enriching the database of 3D structures predicted through in silico studies and derived from the AT11 DNA sequence.

Then, bearing in mind the ultimate goal of developing AT11 derivative sequences, as previously mentioned, we evaluated the molecular interactions and binding sites of AT14 and AT14T in NCL RBD12 and RBD23. For the protein scaffold of NCL RBD12, we used the 3D solution structure determined by NMR spectroscopy (PDB: 2KRR). On the other hand, considering that no solution structure of NCL RBD23 is determined experimentally and available, we predicted that using AlphaFold.

**Figure 6:**
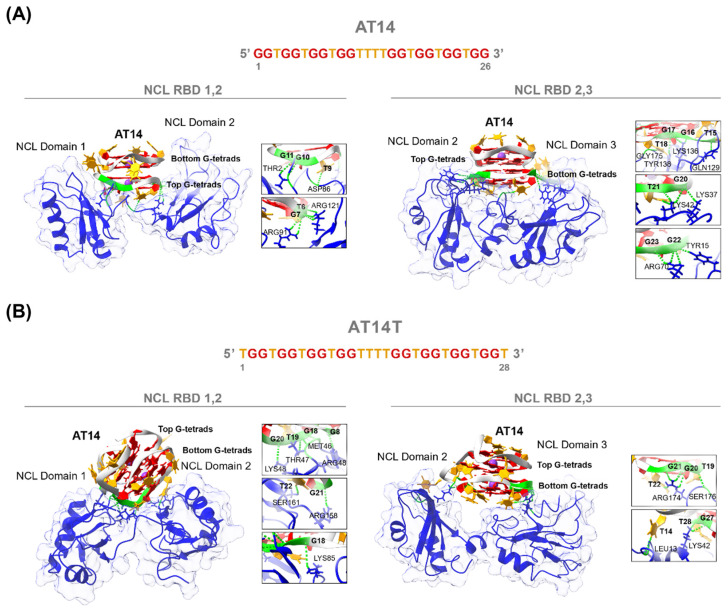
Representative binding mode of (**A**) AT14 and (**B**) AT14T structures with NCL RBD12 and NCL RBD23. The final frames from the MD simulations illustrate the binding pocket located between the two NCL domains, where the DNA G4 structures interact with key residues from both regions. Insets highlight the hydrogen bonds stabilizing the complex. Guanine residues are shown in red, thymine residues in orange, the DNA backbone in light grey, and potassium ions in purple.

First, we docked the structures of AT14 and AT14T into both NCL models. From the ten generated conformers, we selected two based on their binding sites (Figures S7 and S8). After further refinement, we proceeded with MD simulations for the most promising conformer, designated as conformation 1 (Figures S7 and S8, conformation 1). The MD simulations were conducted for 200 ns, and RMSD plots indicated that after an initial equilibration phase, which was longer for the AT14T/NCL RBD23 complex, stability was achieved (Figure S9). Finally, the last frame of each MD trajectory was extracted and analyzed (Figure 6).

The results show that, for both DNA G4 structures (AT14 and AT14T) and both NCL models (RBD12 and RBD23), the binding pocket in the protein is located between the two domains, allowing the DNA G4 structures to establish interactions with both. Furthermore, a similar binding pattern was observed for AT11 and its derivatives [26,28,51], as well as for the RNA G4 structure formed on pre-miRNA-92b, which interacts with NCL RBD12 [50]. These interactions suggest a stable binding mode, where the G4 structures engage key residues from both domains, potentially playing a crucial role in stabilizing the complex and influencing the structural dynamics of NCL upon binding [52]. Moreover, as shown by the insets in Figure 6, several key hydrogen bonds are formed between the two molecules (Tables S5–S8), reinforcing the stability of the complex and suggesting a strong and specific interaction. These molecular interactions may have significant functional implications, potentially modulating NCL’s biological activity and the regulatory mechanisms of NCL [53].

### 3.3. In Cell Studies

#### 3.3.1. Internalization by Confocal Microscopy

**Figure 7:**
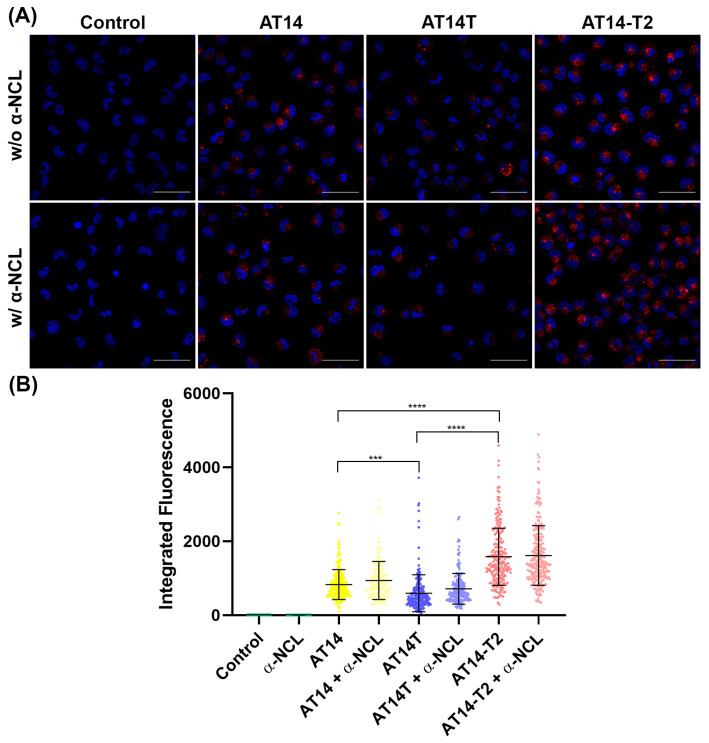
(**A**) Fluorescence confocal microscopy images of H1299 cancer cells incubated with AT14, AT14T, or AT14-T2, without (w/o) or with (w/) the pre-treatment with the anti-nucleolin antibody (α-NCL). The cells were stained with Hoechst 33342 as a nuclear marker (displayed in blue), and an additional red channel was introduced to visualize the sequences’ fluorescence, which is labelled with Cy5. (**B**) Integrated fluorescence values per cell emitted from Cy5-labelled sequences obtained by employing the software ImageJ Version 1.45s (Bethesda, MD, USA) *** *p* < 0.001 and **** *p* < 0.0001; statistical significance was assessed by one-way ANOVA using Tukey’s Multiple Comparisons Test, calculated with GraphPad 9.0.0 software.

The internalization ability of the sequences was then evaluated in the lung cancer cell line H1299 by confocal microscopy. To assess this, incubations were performed with AT14, AT14T, and AT14-T2 sequences, allowing for a comparison of internalization efficiency and the impact of thymine insertion or deletion in the AT14 sequence. As shown in Figure 7, the AT14-T2 sequence exhibited the highest fluorescence levels (mean ± SD = 1582.0 ± 772.7), followed by AT14 (mean ± SD = 829.1 ± 404.4), while AT14T showed the lowest internalization (mean ± SD = 593.9 ± 501.1). These findings suggest that the deletion of two thymine nucleotides in AT14 enhances cellular uptake more effectively than their addition at both the 5′- and 3′-ends. Additionally, the AT14-T2 sequence is the most stable one, as indicated by the CD-melting results (Table S3), which may explain its superior internalization efficiency. Regarding the AS1411 and AT11, they were both demonstrated as being internalized by a nucleolin-overexpressing cell line. Although, besides the proposed sequences being derivatives from AS1411, the results are not completely comparable, since the cell line was different, as previously HeLa cells (a cervical cell line) were used, and the incubation periods were also different, since in this case a 24 h incubation was used, and previously 7 or 3 days were applied (for AS1411 or AT11, respectively) [54,55].

Then, since the studied sequences are derivatives of AS1411, a well-known NCL-binding aptamer [56], experiments were conducted to validate their specificity for NCL. This protein may play a crucial role in target engagement and cellular internalization of the sequences because it is overexpressed on the surface of cancer cells, where it serves as a key target for aptamers such as AS1411 and AT11 [27,56,57]. Therefore, to investigate the role of NCL in the internalization of these sequences, an anti-NCL antibody was used to block surface-expressed NCL, saturating its binding sited and providing insight into whether sequence uptake is mediated by this protein. However, as shown in Figure 7, this blockage did not lead to any significant difference in the quantification of internalized sequences in the acquired confocal microscopy images.

In a previous study, a similar methodology (blocking NCL with an antibody) was used to demonstrate the importance of NCL-mediated internalization of a nanoaggregate functionalized with AS1411 [42]. More specifically, the blockage was performed to confirm that the observed effect on cell viability was specifically due to AS1411 recognition by NCL. Indeed, the cytotoxic effect seen in the cancer cell line was reversed by antibody pre-treatment, with cell viability increasing from approximately 40% to nearly 100% [42]. The differences between our results and those of the previous study could be attributed to variations in incubation times. In our study, we incubated the sequences for 24 h, whereas the previous study used an incubation of 72 h [42]. This suggests that longer incubation times may be necessary to observe significant differences in internalization. This hypothesis is also aligned with other reports, in which it is indicated that NCL-mediated AS1411 internalization occurs at later time points (24–72 h), while initial internalization may be NCL-independent [56,57]. Therefore, in future experiments, longer incubation periods may be required to confirm the role of NCL in sequence internalization.

Since the biophysical experiments demonstrated that the presence of KCl is essential for G4 formation, we also assessed internalization using annealing conditions without salt, where the aptamers do not adopt a G4 structure. In this experiment, as shown in Figure S10, a significant difference in internalization was observed only for the AT14-T2 sequence, suggesting that, in this case, parallel conformation may be necessary for optimal cellular uptake. Additionally, the AT14-T2 sequence is the most stable in the presence of KCl, which could explain the more pronounced difference observed in its internalization when KCl is absent.

#### 3.3.2. Uptake by Flow Cytometry

**Figure 8:**
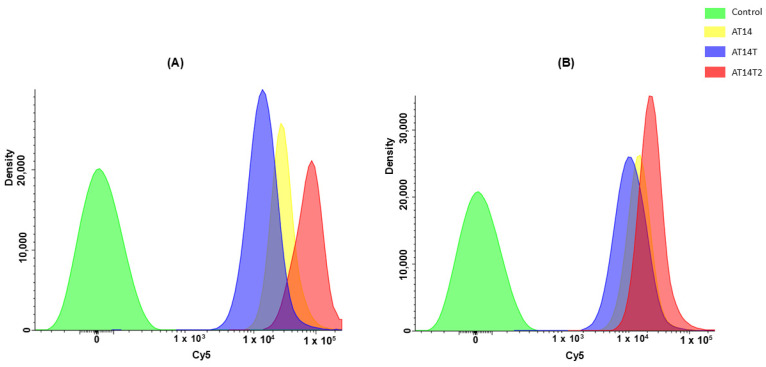
Flow cytometry analysis of H1299 cancer cells incubated with the AS1411 derivatives AT14, AT14T, and AT14-T2, annealed (**A**) with or (**B**) without KCl for 24 h.

Flow cytometry analyses were also performed to evaluate cellular uptake in H1299 cells. All sequences were conjugated with Cy5 to follow its fluorescence and to visualize its cellular uptake. The results presented in Figure 8 indicate that the presence of KCl led to an increase in Cy5 fluorescence intensity across all sequences.

Among the sequences analyzed, AT14T2 exhibited the highest level of cellular internalization in H1299 cells, as evidenced by its stronger fluorescence signal in flow cytometry experiments (Figure 8 and Figure S11). This observation is consistent with CD-melting results, which identified AT14T2 as the most thermally stable sequence, further supporting the hypothesis that structural stability plays a crucial role in cellular uptake.

Additionally, the confocal microscopy images reinforced these findings, as AT14T2 displayed the most intense fluorescence signal among all sequences, confirming its superior internalization efficiency. These results suggest that the stabilization of G4 structures not only influences intracellular uptake mechanisms but also enhances the interaction of these sequences with NCL, potentially increasing their potential internalization in the lung cancer cells.

The results of this study emphasize the significant impact that sequence modifications can have on the formation and stability of G4 structures in DNA aptamers. These modifications reveal the critical interplay between sequence, structure, and function, particularly in how they influence the stability and binding affinity of aptamers for their molecular targets. The ability to fine-tune these sequences provides valuable insights into how specific alterations can enhance or diminish the stability of G4 structures, which directly affects the aptamers’ overall performance and potential applications.

## 4. Conclusions

The study of AS1411’s derivatives aimed to optimize its structural properties and enhance its interaction with NCL while mitigating its polymorphic nature. The G to T substitution, deletion of thymine nucleotides in the central linker, and addition of thymine at both sequence ends, significantly influenced G4 formation, thermal stability, and NCL binding affinity.

Spectroscopic analyses, including CD, FRET-MC, iso-FRET, TDS, and IDS, confirmed that all derivatives retained their ability to form G4 structures and that, notably, sequences with thymine deletions in the central core exhibited increased G4 stability, while thymine additions at the sequence ends resulted in decreased thermal stability. PAGE analysis further supported these findings, indicating that end-modified sequences adopt distinct conformations with altered electrophoretic mobility.

Fluorescence titration assays revealed that structural modifications had an impact on NCL binding affinity. The data reveal that modifications to the thymine linker in AS1411 derivatives significantly affect the affinity toward NCL, with the highest affinity observed for sequences with modifications only on the thymine linker. Interestingly, modifications did not uniformly affect interactions with NCL RBD2,3, suggesting domain-specific binding preferences.

Docking studies and MD simulations provided insights into the conformational dynamics and binding modes of the derivatives to NCL. These findings complement the experimental data, offering a more comprehensive view of the interactions with NCL and their potential for further optimization.

Moreover, confocal microscopy and flow cytometry studies demonstrated that modifications also influenced cellular internalization and uptake, where AT14-T2 proved to have the highest efficiency among the tested derivatives in lung cancer cells, suggesting that structural modifications can enhance cellular targeting.

Overall, this work underscores the intricate balance between aptamer stability, structure, and target affinity. These results lay a foundation for further optimization of AS1411 derivatives, particularly in the context of NCL-targeted cancer therapies.

## Data Availability

Data is contained within the article. The data presented in this study are available on request from the corresponding author.

## Supplementary Materials

The following supporting information can be downloaded at https://www.mdpi.com/article/10.3390/molecules30081673/s1. Table S1. Oligonucleotides used in the work and their properties, Table S2. Ligands used in the experiments, Table S3. *T*_m_ of AS1411 derivatives obtained through CD-melting in K100, Table S4 - Apparent dissociation constants (*K*_D_) of pre-folded Cy5 modified sequences towards NCL RBD1,2 and NCL RBD2,3. The “n” value represents the Hill coefficient, which describes the cooperativity of binding, Table S5. Hydrogen bond interactions between AT14 and NCL RBD1,2 determined using UCSF ChimeraX 1.9, Table S6. Hydrogen bond interactions between AT14 and NCL RBD2,3 determined using UCSF ChimeraX 1.9, Table S8. Hydrogen bond interactions between AT14T and NCL RBD2,3 determined using UCSF ChimeraX 1.9, Figure S1. CD results of AS1411 derivatives at a concentration equivalent to 0.8 absorbance and annealed in LiCaCo, with increasing amounts of KCl (0, 0.1, 1, 10, and 100 mM), Figure S2. Comparison of size exclusion chromatograms of aptamers in K100 buffer, Figure S3. SEC chromatograms fitting and summary of percentages, Figure S4. Fluorescence titration spectra of pre-folded A) Cy5-AT14, B) Cy5-AT14-T1, C) Cy5-AT14-T2, D) Cy5-AT14T, E) Cy5-AT14T-T1, F) Cy5-AT14T-T2 at 100 nM with increasing concentrations of NCL RBD2,3 ranging from 0 to 50 nM, Figure S5. (A) Predicted structure of AT14 G4 (top and side views), obtained from the final snapshot of the 500 ns MD simulation. Guanine residues are highlighted in red while thymine residues are depicted in orange. The backbone is coloured in light grey. (B) B-factor representation of AT14. Colours are ramped from blue over white to red, with blue designating low values and red designating high values. (C) RMSD plot of the 500 ns simulation of AT14 G4 structure. (D) RMSF plot of each nucleotide in the AT14 G4 structure during a 500 ns MD simulation, Figure S6. (A) Predicted structure of AT14T G4 (top and side views), obtained from the final snapshot of the 500 ns MD simulation. Guanine residues are highlighted in red while thymine residues are depicted in orange. The backbone is coloured in light grey. (B) B-factor representation of AT14T. Colours are ramped from blue over white to red, with blue designating low values and red designating high values. (C) RMSD plot of the 500 ns simulation of AT14T G4 structure. (D) RMSF plot of each nucleotide in the AT14T G4 structure during a 500 ns MD simulation, Figure S7. Most relevant molecular docking conformations of AT14-NCL complexes. (A) AT14/NCL 1,2 and (B) AT14/NCL 2,3 in their two most relevant docking conformations. The NCL domains are represented in medium blue, guanines in red, thymines in orange, and the AT14 backbone in light grey. Initial residues of each molecule, protein domains, and G4 tetrads are labelled, Figure S8. Most relevant molecular docking conformations of AT14T-NCL complexes. (A) AT14T/NCL 1,2 and (B) AT14T/NCL 2,3 in their two most relevant docking conformations. The NCL domains are represented in medium blue, guanines in red, thymines in orange, and the AT14T backbone in light grey. Initial residues of each molecule, protein domains, and G4 tetrads are labelled, Figure S9. RMSD plots of the 200 ns simulation of AT14- and AT14T-NCL complexes. (A) AT14/NCL RBD 1,2; (B) AT14/NCL RBD 2,3; (C) AT14T/NCL RBD 1,2, and (D) AT14T/NCL RBD 2,3, Figure S10. (A) Fluorescence confocal microscopy images of H1299 cancer cells incubated with AT14, AT14T or AT14-T2, annealed with (w/) or without (w/o) KCl. The cells were stained with Hoechst 33342 as a nuclear marker (displayed in blue), and an additional red channel was introduced to visualize the sequences’ fluorescence, which is labeled with Cy5. (B) Integrated fluorescence values per cell emitted from Cy5-labelled sequences obtained by employing the software ImageJ in H1299 cells. **** *p* < 0.0001; statistical significance was assessed by one-way ANOVA using Tukey’s multiple comparisons test, calculated with GraphPad software, Figure S11. Comparison of the flow cytometry results of (A) AT14, (B) AT14T, and (C) AT14T2 with (w/) and without (w/o) KCl.
