# Supplementary material for: Exploring the G-Quadruplex Formation of AS1411 Derivatives
Source: Molecules. 2025 Apr 8;30(8):1673. doi: 10.3390/molecules30081673 (PMC12029569; doi:10.3390/molecules30081673)
Supplement: Supplementary file 1 [file molecules-30-01673-s001.zip › molecules-3562961-supplementary.pdf]

SUPPLEMENTARY INFORMATION

Exploring the G-quadruplex formation of AS1411 derivatives

Pedro Lourenço [1], David Moreira [2], André Miranda [2], Jéssica Lopes-Nunes [1], Izamara Maocha [1], Tiago Santos [3], Pedro L. Ferreira [2], Fani Sousa [1], Artur Paiva [4,5,6] and Carla Cruz [2,7,*]

[1] RISE-Health, Department of Medical Sciences, Faculty of Health Sciences, University of Beira Interior, Av. Infante D. Henrique, 6200-506 Covilhã, Portugal; pedro.afonso.amaro.lourenco@ubi.pt (P.L.); jessicalonu@hotmail.com (J.L.-N.); izamaocha@gmail.com (I.M.); fani.sousa@fcsaude.ubi.pt (F.S.)

[2] RISE-Health, Department of Chemistry, Faculty of Sciences, University of Beira Interior, Rua Marquês d'Ávila Bolama, 6201-001 Covilhã, Portugal; david.moreira@ubi.pt (D.M.); andre.miranda@ubi.pt (A.M.) pedroflf@live.com.pt (P.L.F.)

[3] R&Di Division, Instituto de Soldadura e Qualidade, Av. Prof. Dr. Cavaco Silva, 33, Taguspark,

2740-120 Oeiras, Portugal; tiagoaasantos@hotmail.com (T.S.)

[4] Coimbra Institute for Clinical and Biomedical Research (iCBR), Faculty of Medicine, University of Coimbra, Coimbra, Portugal; artur.paiva@chuc.min-saude.pt

[5] Instituto Politécnico de Coimbra, ESTESC-Coimbra Health School, Ciências Biomédicas Laboratoriais, Co-imbra, Portugal

[6] Unidade Funcional de Citometria de Fluxo, Centro Hospitalar E Universitário de Coimbra, Praceta Mota Pinto, 3000-075 Coimbra, Portugal

[7] Department of Chemistry, University of Beira Interior, Rua Marquês d'Ávila e Bolama,

6201-001 Covilhã, Portugal

*Correspondence: carlacruz@fcsaude.ubi.pt

Table S1. Oligonucleotides used in the work and their properties

Name	Sequence 5′ → 3′	MW (g.mol^{-1})	ε (l.mol^{-1}.cm^{-1})	G4H Score	*CF* Factor
AT14	GGTGGTGGTGGTTTTGGTGGTGGTGG	8247.3	248200	1.23	0.22
AT14-T1	GGTGGTGGTGGTTTGGTGGTGGTGG	7943.11	240100	1.28	0.22
AT14-T2	GGTGGTGGTGGTTGGTGGTGGTGG	7638.92	232000	1.33	0.22
AT14T	TGGTGGTGGTGGTTTTGGTGGTGGTGGT	8855.68	264200	1.14	0.18
AT14T-T1	TGGTGGTGGTGGTTTGGTGGTGGTGGT	8551.49	256100	1.19	0.18
AT14T-T2	TGGTGGTGGTGGTTGGTGGTGGTGGT	8247.3	248000	1.23	0.18
[Cy5]-AT14	***[Cy5]***-GGTGGTGGTGGTTTTGGTGGTGGTGG	8887.96	258200	1.23	0.22
[Cy5]-AT14-T1	***[Cy5]***-GGTGGTGGTGGTTTGGTGGTGGTGG	8583.77	250100	1.28	0.22
[Cy5]-AT14-T2	***[Cy5]***-GGTGGTGGTGGTTGGTGGTGGTGG	8279.58	242000	1.33	0.22
[Cy5]-AT14T	***[Cy5]***- TGGTGGTGGTGGTTTTGGTGGTGGTGGT	9496.34	274200	1.14	0.18
[Cy5]-AT14T-T1	***[Cy5]***-TGGTGGTGGTGGTTTGGTGGTGGTGGT	9192.15	266100	1.19	0.18
[Cy5]-AT14T-T2	***[Cy5]***-TGGTGGTGGTGGTTGGTGGTGGTGGT	8887.96	258000	1.23	0.18
ds26	CAATCGGATCGAATTCGATCCGATTG	7970.2	253200	0	0.50
hp2	TCGGTATTGTGTTTCACAATACCGA	7647	241700	0	0.18
22CTA	AGGGCTAGGGCTAGGGCTAGGG	6921.5	220400	1.5	0.55
26CEB	AAGGGTGGGTGTAAGTGTGGGTGGGT	8258.4	265100	1.5	0.27
F21T	**FAM-**GGGTTAGGGTTAGGGTTAGGG-**TAMRA**	8198.7	271180	1.71	-
F22	**FAM-**UGGCCCGUUCGCCCCUCCCGGG	7121.7	194900	-0.909	-
37Q	GGGUUGCGGAGGGUGGGCCUGGGAGGGGU GGUGGCCA-**BHQ1**	12190.9	358200	1.57	0.77

Table S2. Ligands used in the experiments

Name	Formula	MW $(g.mol^{-1})$	CAS
PhenDC3<br>3,3′-[1,10-phenanthroline-2,9-diylbis(carbonylimino)]bis[1-methylquinolinium] 1,1,1-trifluoromethanesulfonate	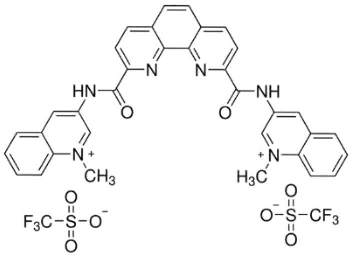<br>$C_{34}H_{26}N_6O_2 \cdot 2CF_3SO_3$	848.75	929895-45-4
Thioflavin T (ThT)	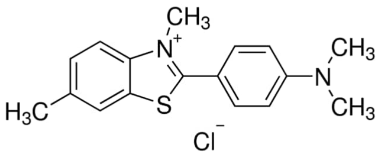<br>$C_{17}H_{19}ClN_2S$	318.86	2390-54-7
N-methyl mesoporphyrin IX (NMM)<br>21H,23H-porphine-2,18-dipropanoic acid, 8,13-diethyl-3,7,12,17,23-pentamethyl	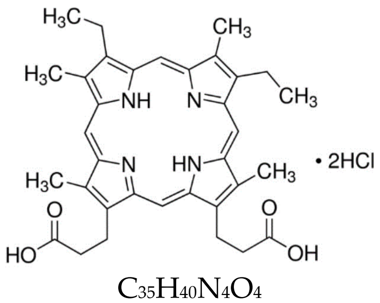<br>$C_{35}H_{40}N_4O_4$	580.72	142234-85-3

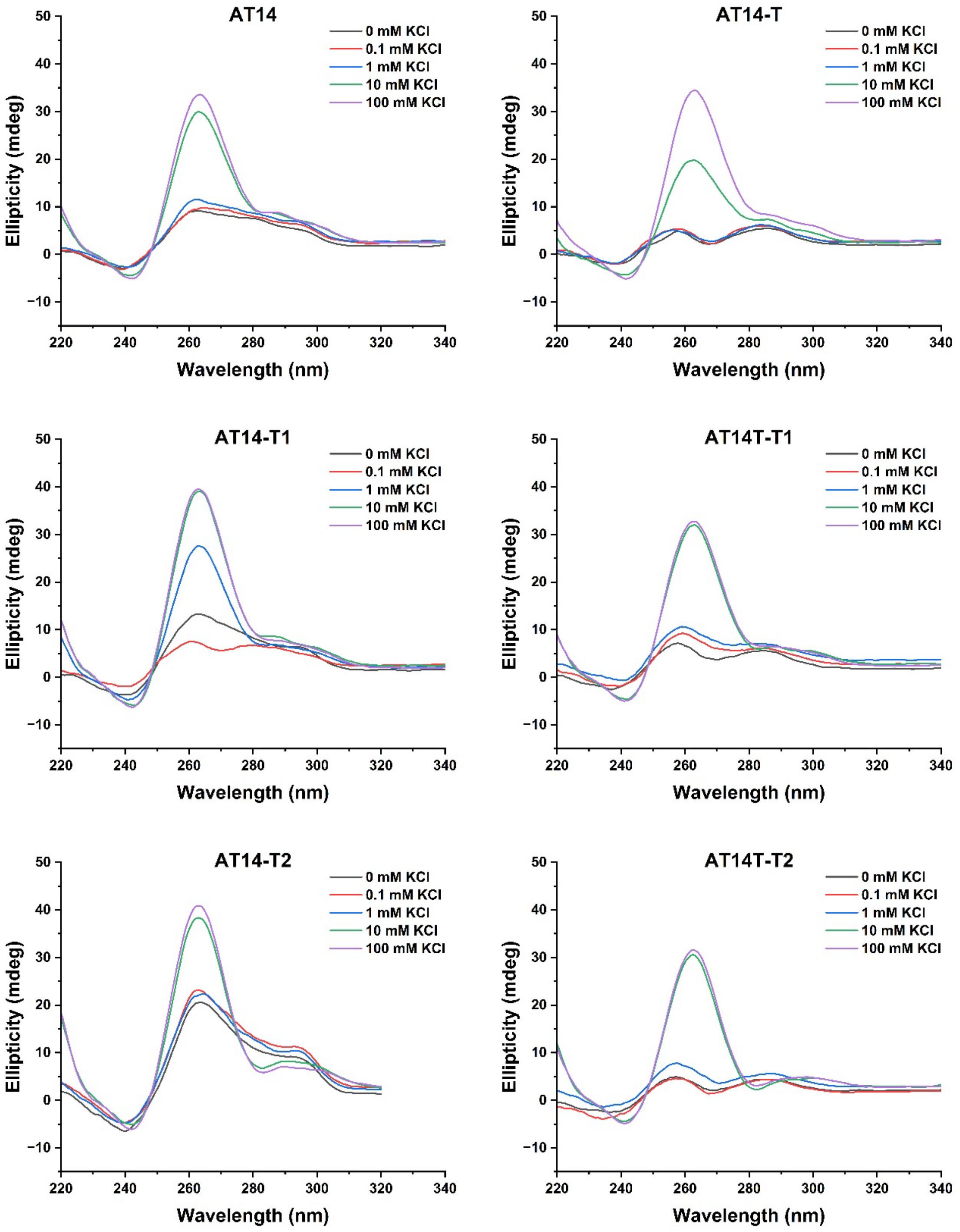

Figure S1. CD results of AS1411 derivatives at a concentration equivalent to 0.8 absorbance and annealed in LiCaCo, with increasing amounts of KCl (0, 0.1, 1, 10, and 100 mM).

Table S3. T_m of AS1411 derivatives obtained through CD-melting in K100.

Sequence	AT14	AT14-T1	AT14-T2	AT14T	AT14T-T1	AT14T-T2
T_m	63.4	68.3	70.2	46.8	54.9	60.0

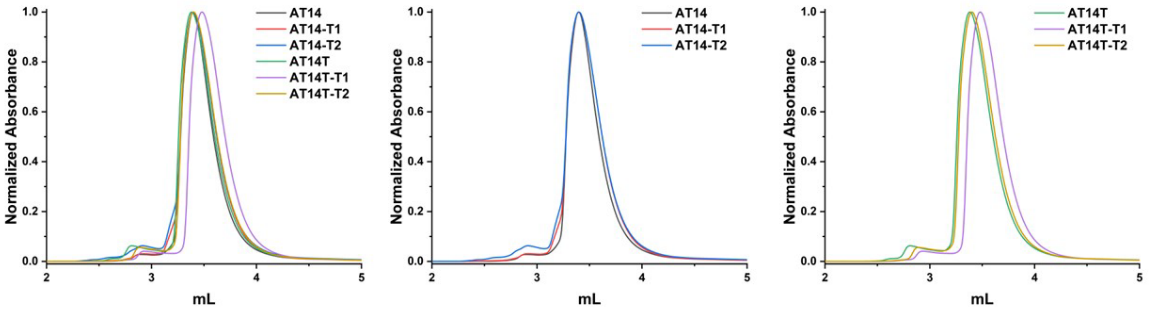

Figure S2. Comparison of size exclusion chromatograms of aptamers in K100 buffer.

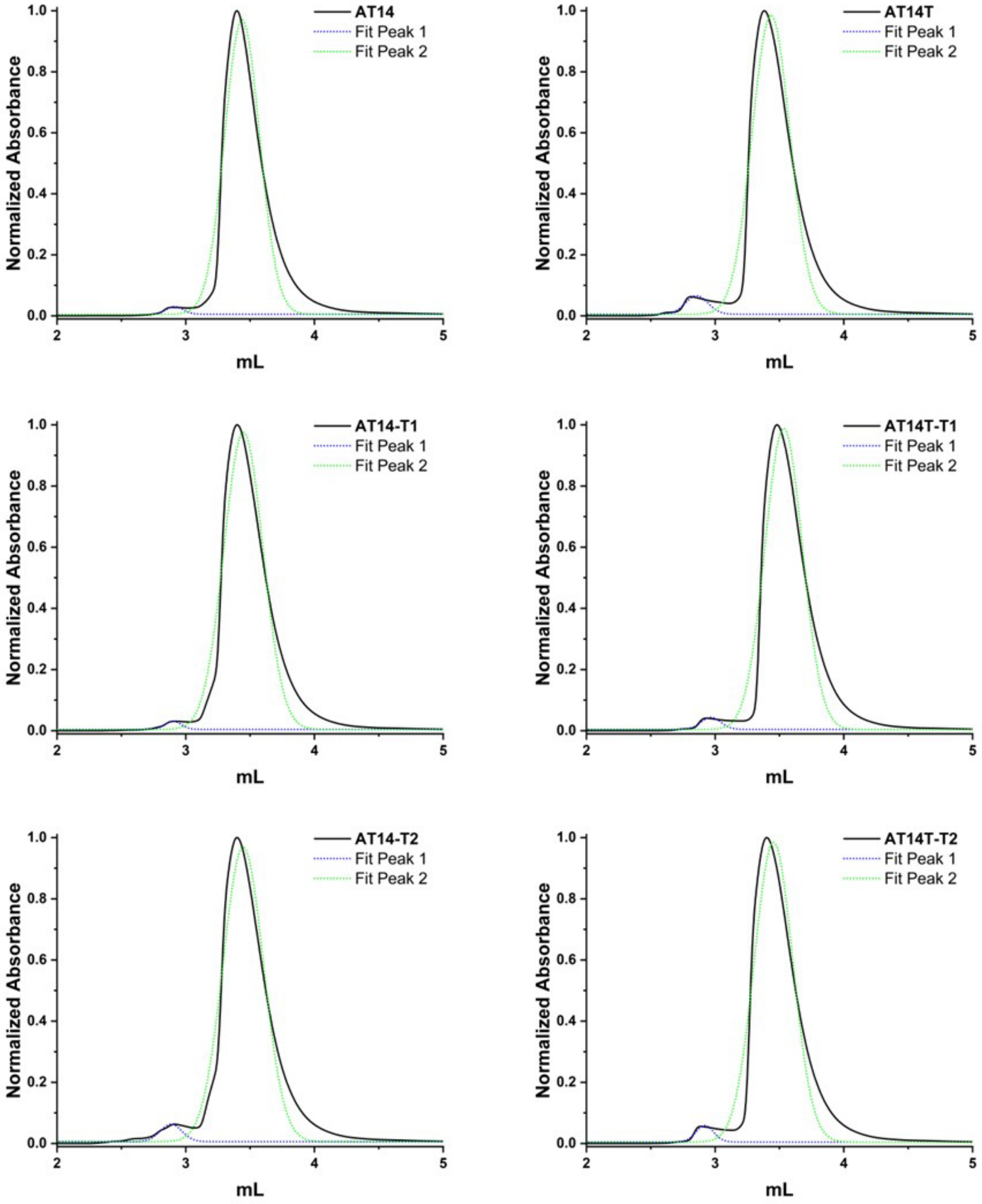

	Peak 1 (%)	Peak 2 (%)
**AT14**	1.224	98.776
**AT14-T1**	1.093	98.907
**AT14-T2**	3.091	96.909
**AT14T**	3.527	96.473
**AT14T-T1**	2.052	97.948
**AT14T-T2**	2.475	97.525

Figure S3. SEC chromatograms fitting and summary of percentages

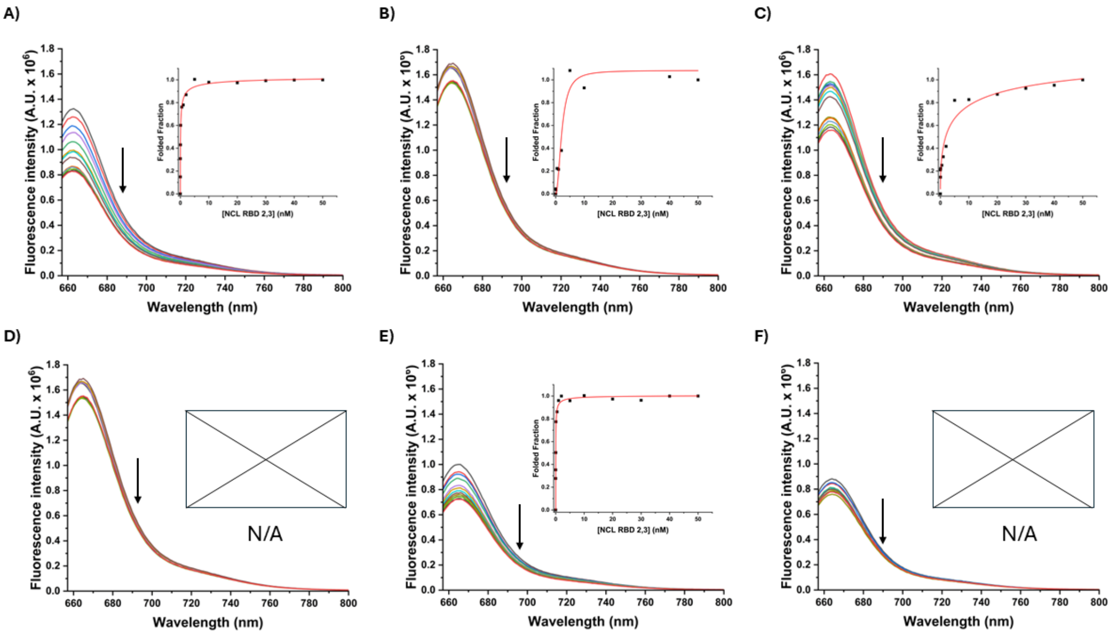

Figure S4. Fluorescence titration spectra of pre-folded **A)** Cy5-AT14, **B)** Cy5-AT14-T1, **C)** Cy5-AT14-T2, **D)** Cy5-AT14T, E) Cy5-AT14T-T1, F) Cy5-AT14T-T2 at 100 nM with increasing concentrations of NCL RBD2,3 ranging from 0 to 50 nM.

Table S4 - Apparent dissociation constants (K_D) of pre-folded Cy5 modified sequences towards NCL RBD1,2 and NCL RBD2,3. The "n" value represents the Hill coefficient, which describes the cooperativity of binding.

**Sequence**	**NCL RBD1,2**		**NCL RBD2,3**	
	**K_D (nM)**	**n**	**K_D (nM)**	**n**
**AT14**	0.46 ± 0.12	0.37	0.08 ± 0.01	0.54
**AT14T**	8.34 ± 13.23	0.31	*	
**AT14-T1**	1.40 ± 4.47	0.20	2.16 ± 0.37	2.08
**AT14T-T1**	3.39 ± 0.58	0.24	0.03 ± 0.01	0.67
**AT14-T2**	0.01 ± 0.01	0.33	5.37 ± 7.21	0.49
**AT14T-T2**	1.48 ± 0.20	1.89	*	

* It was not possible to fit the Hill model.

In cases where the standard deviation (SD) exceeds the K_D value, this reflects variability in binding measurements, likely due to weak or inconsistent interactions.

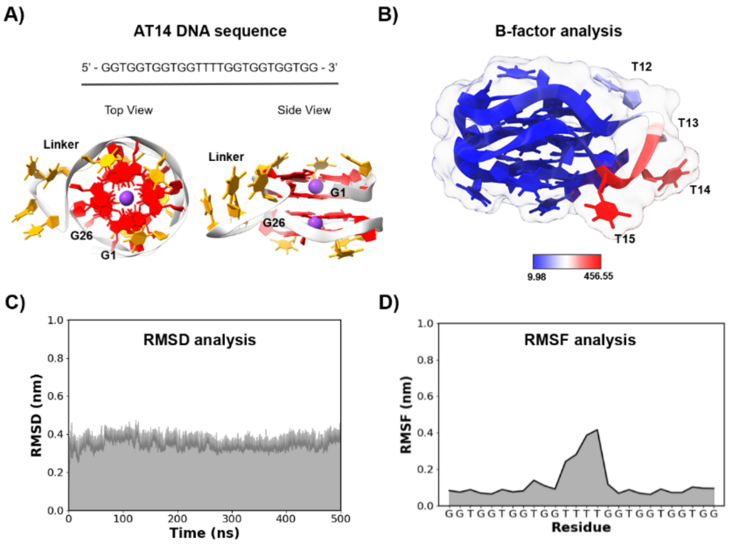

Figure S5. (A) Predicted structure of AT14 G4 (top and side views), obtained from the final snapshot of the 500 ns MD simulation. Guanine residues are highlighted in red while thymine residues are depicted in orange. The backbone is coloured in light grey. **(B)** B-factor representation of AT14. Colours are ramped from blue over white to red, with blue designating low values and red designating high values. (C) RMSD plot of the 500 ns simulation of AT14 G4 structure. **(D)** RMSF plot of each nucleotide in the AT14 G4 structure during a 500 ns MD simulation.

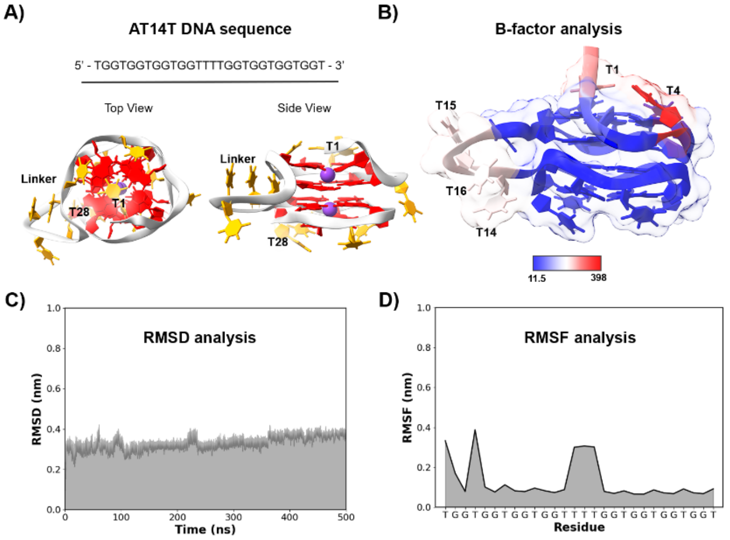

Figure S6. (A) Predicted structure of AT14T G4 (top and side views), obtained from the final snapshot of the 500 ns MD simulation. Guanine residues are highlighted in red while thymine

residues are depicted in orange. The backbone is coloured in light grey. **(B)** B-factor representation of AT14T. Colours are ramped from blue over white to red, with blue designating low values and red designating high values. (C) RMSD plot of the 500 ns simulation of AT14T G4 structure. **(D)** RMSF plot of each nucleotide in the AT14T G4 structure during a 500 ns MD simulation.

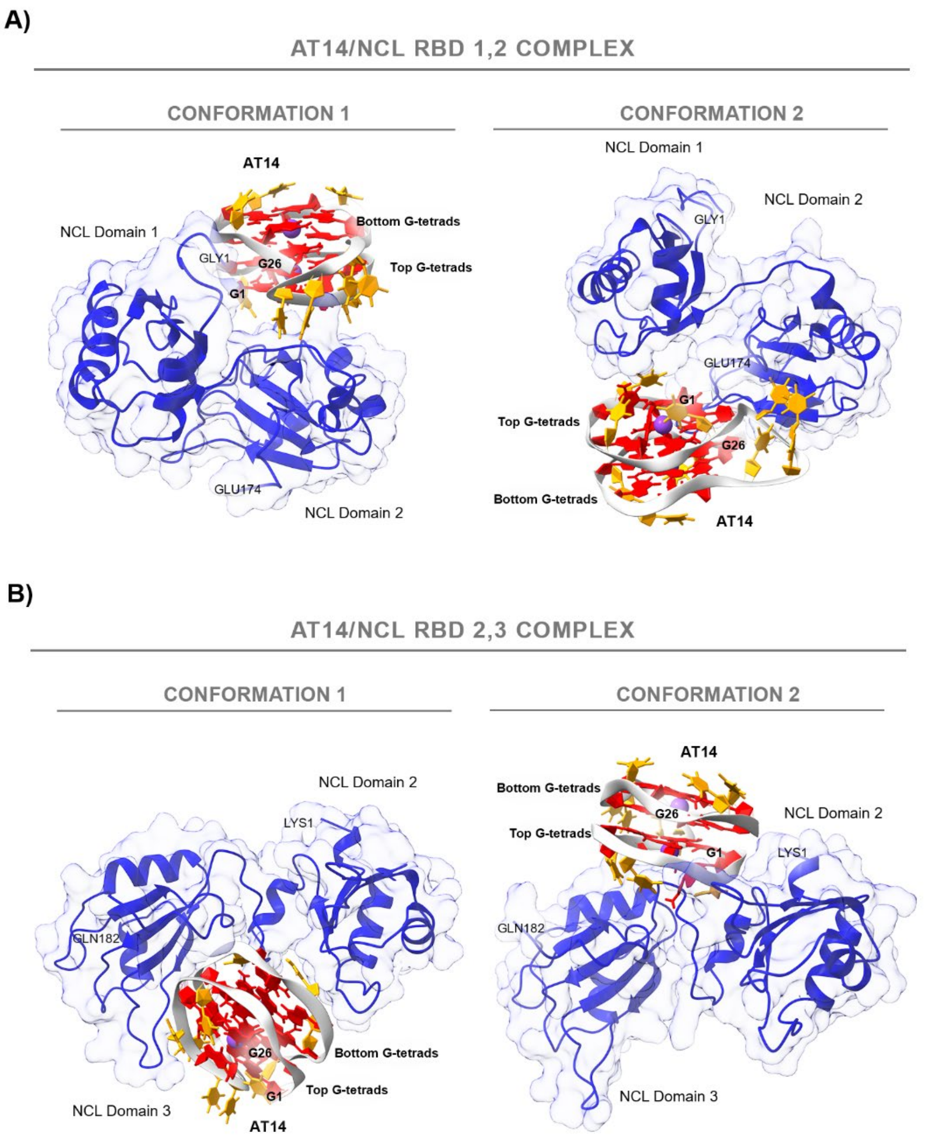

Figure S7. Most relevant molecular docking conformations of AT14-NCL complexes. **(A)** AT14/NCL 1,2 and **(B)** AT14/NCL 2,3 in their two most relevant docking conformations. The NCL domains are represented in medium blue, guanines in red, thymines in orange, and the AT14 backbone in light grey. Initial residues of each molecule, protein domains, and G4 tetrads are labeled.

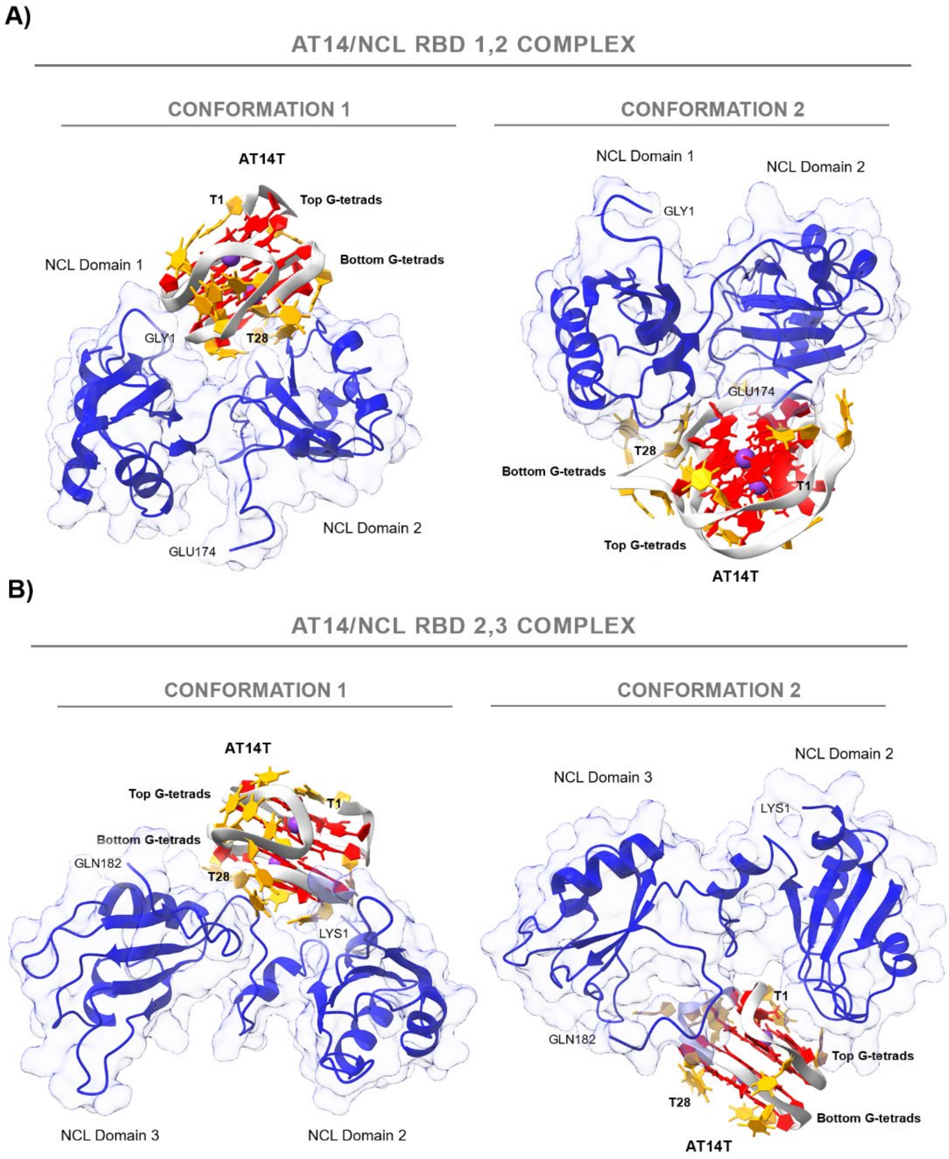

Figure S8. Most relevant molecular docking conformations of AT14T-NCL complexes. **(A)** AT14T/NCL 1,2 and **(B)** AT14T/NCL 2,3 in their two most relevant docking conformations. The NCL domains are represented in medium blue, guanines in red, thymines in orange, and the AT14T backbone in light grey. Initial residues of each molecule, protein domains, and G4 tetrads are labeled.

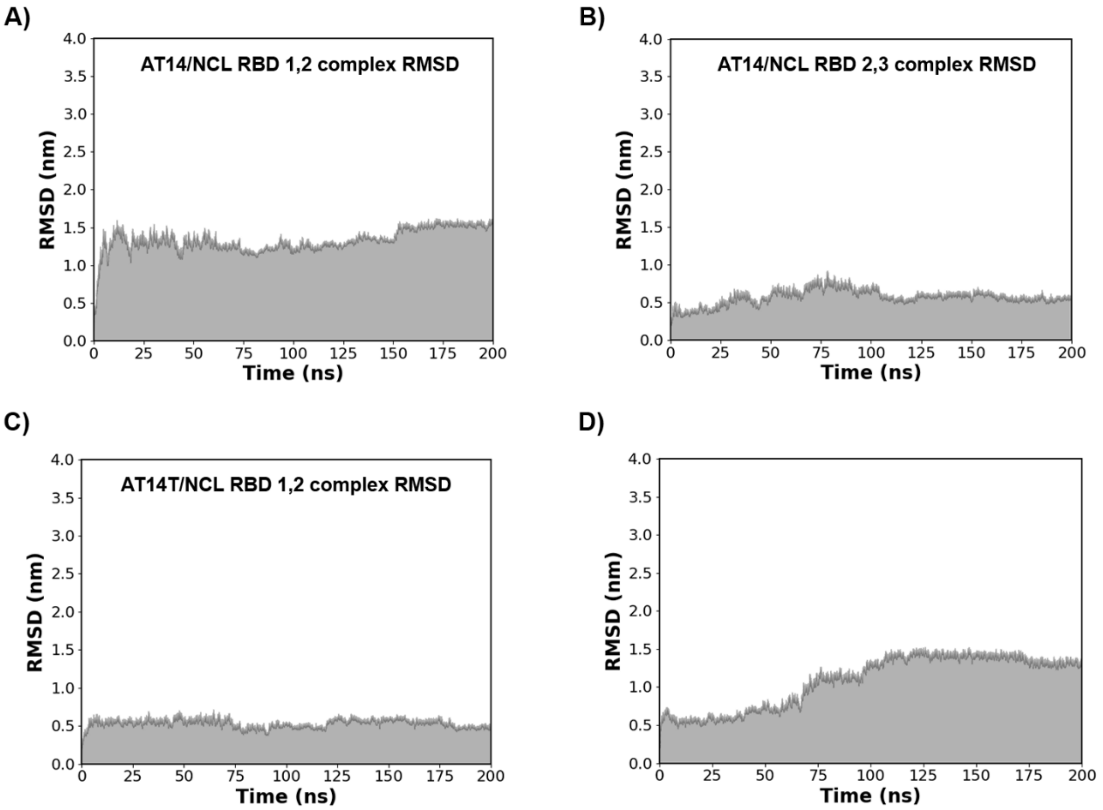

Figure S9. RMSD plots of the 200 ns simulation of AT14- and AT14T-NCL complexes. **(A)** AT14/NCL RBD 1,2; **(B)** AT14/NCL RBD 2,3; **(C)** AT14T/NCL RBD 1,2, and **(D)** AT14T/NCL RBD 2,3.

Table S5. Hydrogen bond interactions between AT14 and NCL RBD1,2 determined using UCSF ChimeraX 1.9.

Hydrogen Bond	Donor Residue	Donor Atom	Acceptor Residue	Acceptor Atom	Hydrogen Atom	D..A Distance (Å)	D-H..A Distance (Å)
1	T9	N3	ASP86	O	H3	2.745	1.825
2	THR2	N	G10	O3'	H	2.954	2.032
3	THR2	OG1	G11	O1P	HG1	3.046	2.124
4	ARG91	NH1	G7	O2P	HH12	2.662	1.680
5	ARG91	NH2	G7	O2P	HH22	2.990	2.141
6	ARG121	NE	G7	O1P	HE	2.863	1.921
7	ARG121	NH2	T6	O1P	HH22	2.737	1.778
8	ARG121	NH2	G7	O1P	HH21	3.038	2.092

Table S6. Hydrogen bond interactions between AT14 and NCL RBD2,3 determined using UCSF ChimeraX 1.9.

Hydrogen Bond	Donor Residue	Donor Atom	Acceptor Residue	Acceptor Atom	Hydrogen Atom	D..A Distance (Å)	D-H..A Distance (Å)
1	TYR15	OH	G22	O1P	HH	2.602	1.750
2	LYS37	NZ	G20	O1P	HZ3	3.223	2.361
3	LYS42	NZ	G20	O1P	HZ1	2.996	1.997
4	LYS42	NZ	T21	O2	HZ2	2.741	1.879
5	LYS42	NZ	T21	O2P	HZ3	2.791	1.823
6	ARG70	NH1	G22	O2P	HH12	2.746	1.815
7	ARG70	NH2	G22	O2P	HH22	2.896	1.937
8	ARG70	NH2	G23	O1P	HH21	2.782	1.929
9	GLN129	NE2	T15	O1P	HE21	2.965	2.007
10	LYS136	NZ	G16	O2P	HZ1	2.785	1.945
11	LYS136	NZ	G17	O1P	HZ2	2.659	1.684
12	TYR138	OH	G17	O2P	HH	2.634	1.677
13	GLY175	N	T18	O2	H	2.780	1.928

Table S7. Hydrogen bond interactions between AT14T and NCL RBD1,2 determined using UCSF ChimeraX 1.9.

Hydrogen Bond	Donor Residue	Donor Atom	Acceptor Residue	Acceptor Atom	Hydrogen Atom	D..A Distance (Å)	D-H..A Distance (Å)
1	MET46	N	G18	O3'	H	3.227	2.232
2	THR47	N	T19	O1P	H	2.814	1.835
3	THR47	OG1	T19	O1P	HG1	2.694	1.742
4	ARG48	NH1	G8	O1P	HH12	2.865	1.885
5	LYS49	NZ	G20	O1P	HZ3	2.884	1.905
6	LYS85	NZ	G18	O2P	HZ3	2.858	1.957
7	ARG158	NH2	G21	O2P	HH21	2.84	1.845
8	SER161	OG	T22	O4	HG	2.65	1.737

Table S8. Hydrogen bond interactions between AT14T and NCL RBD2,3 determined using UCSF ChimeraX 1.9.

Hydrogen Bond	Donor Residue	Donor Atom	Acceptor Residue	Acceptor Atom	Hydrogen Atom	D..A Distance (Å)	D-H..A Distance (Å)
1	T14	N3	LEU13	O	H3	3.021	2.055
2	LYS42	NZ	G27	O2P	HZ2	2.995	1.998
3	LYS42	NZ	T28	O2P	HZ3	2.961	2.115
4	ARG174	NE	G21	O2P	HE	3.134	2.247
5	ARG174	NH1	T22	O2	HH11	2.819	1.915
6	ARG174	NH2	G21	O2P	HH21	3.131	2.177
7	ARG174	NH2	G21	O5'	HH21	3.207	2.461
8	SER176	N	T19	O3'	H	2.798	1.879

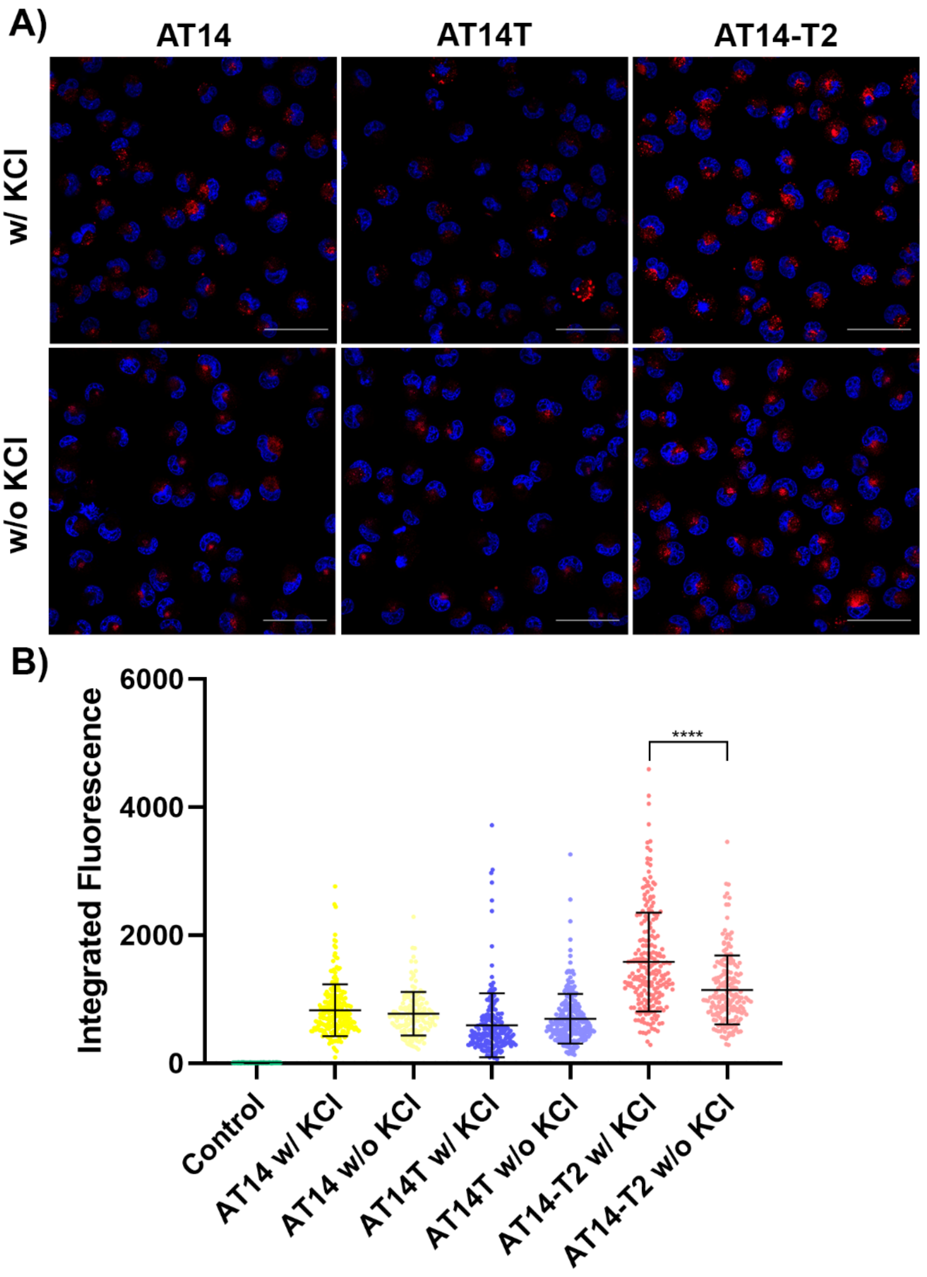

Figure S10. A) Fluorescence confocal microscopy images of H1299 cancer cells incubated with AT14, AT14T or AT14-T2, annealed with (w/) or without (w/o) KCl. The cells were stained with Hoechst 33342 as a nuclear marker (displayed in blue), and an additional red channel was introduced to visualize the sequences' fluorescence, which is labeled with Cy5. **B)** Integrated fluorescence values per cell emitted from Cy5-labelled sequences obtained by employing the software ImageJ in H1299 cells. ****$p < 0.0001$; statistical significance was

assessed by one-way ANOVA using Tukey's multiple comparisons test, calculated with GraphPad software.

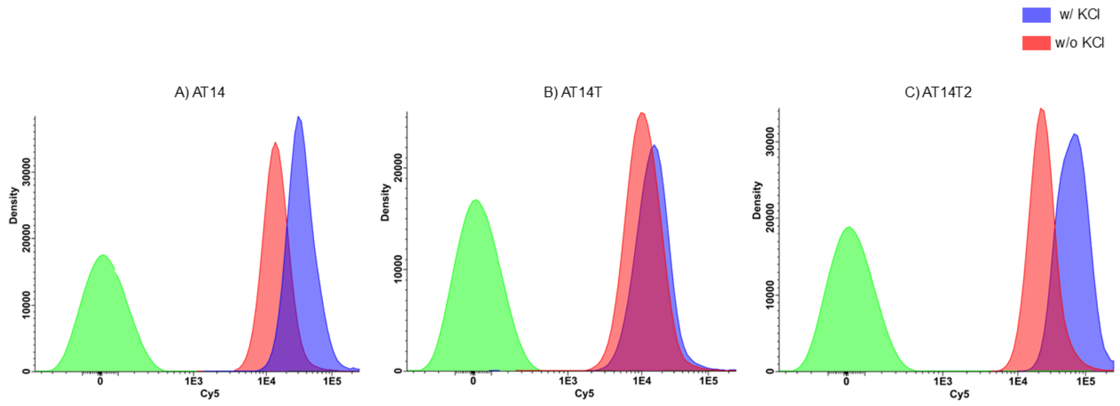

Figure S11. Comparison of the flow cytometry results of **A)** AT14, **B)** AT14T, and **C)** AT14T2 with (w/) and without (w/o) KCl.
